# Exploring the Enhanced Antiproliferative Activity of Turmeric Oil and 6-Mercaptopurine in a Combined Nano-Particulate System Formulation

**DOI:** 10.3390/pharmaceutics15071901

**Published:** 2023-07-07

**Authors:** Tarek A. Ahmed, Ehab M. M. Ali, Abdulaziz A. Kalantan, Alshaimaa M. Almehmady, Khalid M. El-Say

**Affiliations:** 1 Department of Pharmaceutics, Faculty of Pharmacy, King Abdulaziz University, P.O. Box 80260, Jeddah 21589, Saudi Arabia; amnalmehmady@kau.edu.sa; 2 Department of Biochemistry, Faculty of Science, King Abdulaziz University, Jeddah 21589, Saudi Arabia; emali@kau.edu.sa (E.M.M.A.); aakalantan@kau.edu.sa (A.A.K.)

**Keywords:** turmeric oil, self-nanoemulsifying drug delivery system, 6-mercaptopurine, antiproliferative activity, HepG2, MCF-7

## Abstract

6-Mercaptopurine (6-MP) is a chemotherapeutic agent with inadequate efficacy due to its poor aqueous solubility and limited bioavailability. Turmeric oil is a naturally occurring bioactive substance obtained from the rhizomes of *Curcuma longa Linn* that has well-known antiproliferative activities. The aim of this study was to develop a 6-MP-loaded turmeric oil-based self-nanoemulsifying drug delivery system (SNEDDS) to improve the anticancer activity of 6-MP. Turmeric oil was extracted and used in a range of 15–25% to develop SNEDDS formulations utilizing tween 80 and dimethyl sulfoxide as the surfactant and cosurfactant, respectively. The size, charge, and effect of the formulations on the viability against HepG2 and MCF-7 cell models, as well as the apoptosis and cell cycle, were analyzed. The prepared SNEDDS formulations were in the size range of 425.7 ± 7.4–303.6 ± 19.3 nm, using a polydispersity index of 0.429–0.692 and electronegative surface charges. Moreover, 6-MP-loaded SNEDDS with 15% turmeric oil content (F1) showed smaller particle sizes and a noticeable antiproliferative activity against both cell line models. Also, F1 showed a higher rate of late apoptosis than the pure drug and the corresponding non-medicated formulation. A morphological study revealed significant changes in the HepG2 cells compared to untreated cells. More cells halted in the S phase, and a marked decrease in the proportions of cells in the G1/G0 phase was observed when using SNEDDS formulation compared to pure drug. Thus, SNEDDS formulation is a promising drug delivery system for improving the antiproliferative activity of 6-MP, especially when turmeric oil is incorporated.

## 1. Introduction

Turmeric oil is a naturally extracted oil obtained from the rhizomes of turmeric (*Curcuma longa Linn*). Turmeric oil (TO) contains several bioactive constituents that are responsible for significant pharmacological activities. The major constituents include curcumin, curcuminoids, aromatic–turmerone, α-turmerone, β-turmerone, α-atlantone, ar-curcumene, γ-curcumene, curlone, p-cymene, bisabolanes, guaianes, germacranes, caranes, elemanes, spironolactones, selinanes, santalanes, and caryophyllanes [1]. Turmeric oil is known to have many medical activities, such as anti-inflammatory [2], antimicrobial [3], antidiabetic [4], analgesic, and antiproliferative effects [1]. A report by Hastak et al. showed that turmeric oil slowed down cytogenetic damage in vitro in patients with submucous pre-cancerous lesions by preventing micronucleated cell growth in lymphocytes [5]. Moreover, a prior report showed that turmeric oil hinders tobacco extract’s in vitro mutagenicity and prevents mutagens’ microsomal activation [6]. Another study reported that turmeric oil stimulated apoptosis in A431 human cancer cell line and stopped cell proliferation [7]. In addition, induction of apoptotic modulators, such as caspase-3 and caspase 8, Bcl-2, Bax, and NF-ЌB, was observed in the case of benign prostatic hyperplasia in animal models [8]. Furthermore, turmeric oil stopped the growth of the mammalian colon cancer cell line (HT-116 and HT-29) [9]. In a clinical trial for patients with hepatic cancer, TO improved average survival times [10,11].

6-Mercaptopurine (6-MP) is a purine analogue drug that contains the thiol (-SH) group, which is known for its antiproliferative and immunosuppressive activities [12]. It has been reported to inhibit cell growth via various mechanisms: for example, 6-MP inhibits DNA chain elongation by blocking DNA polymerase action. Moreover, 6-MP stops ATP generation and induces programmed cell death, limiting cell proliferation and tumor progression in the body [13]. In addition, 6-MP disrupts DNA replication and RNA transcription [14]. Moreover, 6-MP is characterized by its small molecular weight (152.177 g/mol), low water solubility (0.22 mg/mL), and relatively low absorption rate (16–20%). The drug suffers from a short biological half-life and many side effects [15]. Accordingly, new formulation strategies are required to enhance the drug’s anti-cancer activities and reduce its side effects.

Nanotechnology is considered to be a successful approach to drug delivery that influences pharmacological activity, especially in hydrophobic drugs. Nanotechnology-based drug delivery systems offer an alternative way to deliver active pharmaceutical ingredients with improved bioavailability and therapeutic activity [16]. A self-nano emulsifying drug delivery system (SNEDDS) is a homogenous mixture of oils, surfactants, and cosurfactants that has been reported to enhance the bioavailability of many pharmaceutical ingredients by improving solubility and permeability [17]. SNEDDS will spontaneously emulsify and produce o/w droplets in the 20–200 nm size range after dispersion in an aqueous environment through gentle agitation [18]. Additionally, SNEDDS have been linked to increased transcellular permeability due to their capacity to increase the lipid fluidity of enterocyte membranes and inhibit efflux pumps, thus enhancing bioavailability and pharmacological activity [17].

In the current work, we are investigating the enhanced antiproliferative activity of turmeric oil in the form of SNEDDS and 6-MP on two different cell line models, namely HepG2 and MCF-7. Drug-loaded SNEDDS formulations were prepared, characterized, and tested in cells to explore the cytotoxicity criteria, including cell viability, apoptosis, and the changes in cell cycle stages.

## 2. Materials and Methods

### 2.1. Materials

6-Mercaptopurine (6-MP), tween^®^ 80, and dimethyl sulfoxide (DMSO) were purchased from Sigma-Aldrich Company Ltd. (St. Louis, MO, USA). Thiazolyl blue tetrazolium bromide (MTT), trypan blue, and propidium iodide were procured from Merck and Co., Inc. (West Point, PA, USA). Dulbecco’s Modified Eagle’s Medium (high glucose), fetal bovine serum, HepG2 and MCF-7 cell cultures, and 5000 U/mL of penicillin–streptomycin antibiotics were obtained from Thermo Fisher Scientific (Waltham, MA, USA). The Annexin V-FITC Apoptosis Kit was purchased from Creative Biolabs (Shirley, NY, USA).

### 2.2. Development of Turmeric Oil Based SNEDDS

The dried rhizomes of Curcuma longa “Turmeric” (Family Zingiberaceae) were purchased from a local supplier in Jeddah, Saudi Arabia, and the oil was extracted using the process described in our most recently published work [19].

The extracted turmeric oil was used to develop six different medicated SNEDDS formulations, and it utilized Tween 80 as a surfactant and DMSO as a co-surfactant. Details of the composition of the prepared SNEDDS formulations are illustrated in Table 1. In short, the specified amount of each component (oil, tween, and DMSO) was precisely weighed and placed in a screw cap vial that was subjected to vortex mixing to prepare a homogenous mixture. Non-medicated SNEDDS formulations (F7–F12), which contained the same components but lacked the drug, were also prepared for comparative study.

### 2.3. Characterization of the Prepared SNEDDS Formulations

The prepared medicated SNEDDS formulations were characterized for particle size, polydispersity index (PDI), and zeta potential using Malvern Zetasizer Nano ZSP, Malvern Panalytical Ltd. (Malvern, UK). Before measurement, each SNEDDS formulation was diluted 10-fold with distilled water. All measurements were performed at room temperature (25 °C). The number of runs, scans, voltages, and attenuation settings for each sample was automatically adjusted. Each formulation was measured in triplicate, and the average figure was considered. Data were analyzed using Malvern Zetasizer software version 7.12.

### 2.4. Solubility of 6-MP in the Prepared SNEDDS Formulations

The solubility of 6-MP in the prepared SNEDDS formulations was evaluated by adding an excess amount of the drug to the known volume (3 mL) of the formulation in a screw-capped glass vial. The prepared glass vials were mixed correctly using a vortex mixer (Velp Scientifica, ZX3) and kept in a shaking water bath (GFL Corporation, type 1083, Burgwedel, Germany) at 25 ± 0.5 °C for 3 days. The content of each vial was filtered using a 0.45 μm syringe filter, suitably diluted with methanol, and subjected to spectrophotometric analysis at 332 nm to determine the amount of 6-MP. All trials were performed thrice, and results were expressed in terms of mg/mL as the mean ± standard deviation (SD).

### 2.5. Evaluation of Cell Viability of the Treated HepG2 and MCF-7

HepG2 and MCF-7 indicated hepatocellular carcinoma hepatoma G2 (HepG2) and breast cancer cell lines (Michigan Cancer Foundation-7) (MCF-7) as examples of attached human cell lines that were available in our laboratory. HepG2 and MCF-7 were obtained from the Tissue Culture Unit, thr Department of Biochemistry, the Faculty of Science, King Abdulaziz University. The selected human cell lines were grown in Dulbecco’s Modified Eagle’s Medium with 10% fetal bovine serum at 37 °C in a CO_2_ incubator. After 70–90% confluence, 5 mL of 0.25% trypsin was added to aid cell detachment. The cells were seeded in a 96-well plate, and counted using trypan blue, and the concentration of the cell was set at 10^5^/mL. The plate was filled with 100 mL of medium in each well and incubated for 24 h. The media in each well was changed to include media with various 6-MP concentrations and formalized 6-MP with and without curcumin. The concentrations of 6-MP were adjusted at 50, 25, 12.5, 6.25, and 3.125 µg/mL (as well as equivalent volumes of corresponding non-medicated formulations). There were three repetitions of each concentration. After 48 h of incubation at 37 °C, the cells were washed with phosphate buffer, and 100 mL of the 0.5 mg/mL MTT was added to each well. The 96-well plate was incubated for 4 h at 37 °C in the dark. After removing the MTT and replacing it with 100 mL of DMSO, the plate was allowed to rest for 15 min. Using an ELISA reader, the absorbance was measured at 595 nm (Bio-RAD microplate reader, Japan) [20,21].

The changes in the morphologies of the studied cells following treatment with the prepared formulations were investigated using Nikon ECLIPSE Ti-S (Japan).

### 2.6. Assessment of Apoptosis via Annexin V

Following the transplantation of the cells for 24 h in a T-75 flask, which utilized the same condition stated in the preceding section, the cells were transferred into a Falcon tube and counted. Next, 2 mL of medium containing 2 × 10^5^ cells was added to each well of a six-well plate. The plate was incubated for 24 h to promote cell proliferation. Subsequently, the medium was changed to a medium containing 12.5 mg/ mL 6-MP, 6-MP with curcumin (F1 and F4), and the corresponding non-medicated formulations. After 24 h, 0.5 mL of 0.25% trypsin was added to each well to detach the cells. The plate was kept at 37 °C for 5 min. The content of each well was transferred into Falcon tubes and centrifuged. The obtained pellets were washed twice with phosphate buffer, re-suspended in 400 mL of binding buffer, and complemented with 25 mL of Annexin V-FITC/propidium iodide (PI). The tubes were thoroughly mixed and incubated at room temperature for 5 min in a dark area. The cells were analyzed via flow cytometry provided by Applied Biosystems, Thermo Fisher Scientific (Waltham, MA, USA) [22].

### 2.7. Assessment of Cell Cycle

In a 6-well plate, the studied cells were cultivated for 24 h, as described above, though each well was filled with 1 × 10^6^ cells. A fresh medium containing the same amount of free 6-MP and the studied formulations (F1, F4, and the corresponding non-medicated formulations) was added and replaced after 48 h. The cells in each well were collected in tubes and washed two times with phosphate buffer. The studied cells were suspended in 300 mL of phosphate buffer, 0.7 mL of 100% ethanol was gradually added, and the tubes were maintained at −4 °C for about one hour. The cells were subjected to centrifugation, and the pelleted cells were then mixed with 250 µL of a 50 μg/mL propidium iodide solution and 100 µL of phosphate buffer. Propidium iodide was used to bind with the DNA of the studied cells at each phase of the cell cycle. The tubes were kept in a dark place for about 1 h and, finally, analyzed through flow cytometry (BD FACSCanto^TM^ II) to determine the proportion of cells in each cell-cycle stage [23].

### 2.8. Statistical Analysis

Results are presented as mean ± SD to facilitate formulation characterization and investigation of the viability of the treated cells. GraphPad Prism Software (version 9.0) was used to compute the drug IC_50_ and statistically analyze all obtained data. The flow cytometry software was used to automatically determine the percentage of cells in each phase and the quantity of necrotic and apoptotic cells. The t-test was performed to determine the significance of treatment differences.

## 3. Results and Discussion

### 3.1. Development and Characterization of Turmeric Oil-Based SNEDDS

In this study, Tween 80 was selected as a surfactant to develop 6-MP-loaded curcumin-based SNEDDS formulation, since it is a non-ionic and biocompatible substance that was successfully used in our previously published works with different oils to develop SNEDDS with the lowest possible globule size [19]. Also, DMSO was selected as a cosurfactant, since it is considered to be a safe substance with very low acute and chronic toxicity for animal, plant, and aquatic life, even at high concentrations [24]. DMSO is widely used in cell culture. It has been reported that it could not modify culture viability in a concentration of up to 10% [25]. DMSO was used as a cosurfactant, tween was used as a surfactant, and coconut oil was used to develop citral-loaded SNEDDS, and the authors assessed their anti-proliferative activities against colorectal cancer cells [26].

Characterization results for the prepared medicated SNEDDS formulations revealed that the maximum drug loads were obtained for F1 and F4 at 20 mg/mL and 19.4 mg/mL, respectively, as depicted in Table 1. This behavior could be attributed to the high concentration of DMSO in these formulations. The particle size range for the prepared medicated SNEDDS formulations was 303.6 ± 19.3–425.7 ± 7.4 nm.

**Figure 1:**
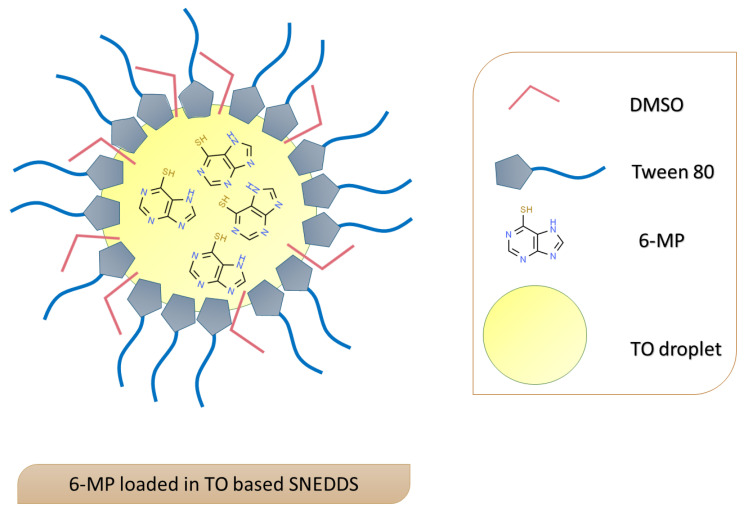
Scheme representing 6-MP loaded turmeric oil-based SNEDDS.

As the concentration of oil increased and the concentration of the cosurfactant decreased, the particle size of the prepared SNEDDS increased. This behavior could be attributed to the availability of more surfactant and cosurfactant molecules at low oil concentrations, which stabilize the oil/water interface and promote emulsification, as previously reported [27,28]. The obtained PDI results were located in the range 0.429–0.692, which indicates good uniformity of size, as previously reported [29]. Zeta potential analysis of the studied formulations, which reflects the electro-kinetic potential present in the surface of the particles, indicated the electronegative nature of the surface charges. These negative charges on the surface could be attributed to the presence of the (-SH) and (-OH) groups in the drug and the incorporated oil, which ionized in the aqueous medium. A schematic representation of the prepared drug-loaded SNEDDS is illustrated in Figure 1.

### 3.2. Effect of the Prepared Formulation on the Anti-Tumor Activity and Cell Morphology

**Figure 2:**
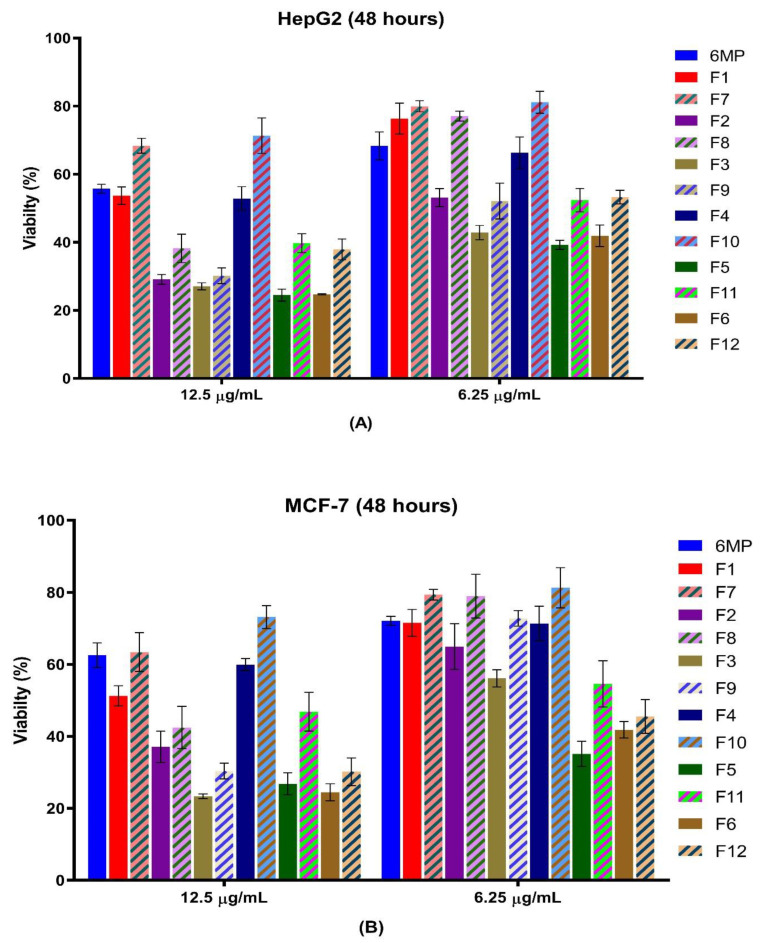
Viability percentage of HepG2 and MCF-7 cells after treatment with 12.5 µg/mL and 6.25 µg/mL of free 6-MP, medicated (full colored bars) and the corresponding non-medicated (striped colored bars) SNEDDS formulations. (**A**), HepG2 cells; (**B**), MCF-7 cells.

After 48 h of treatment with the prepared medicated formulations and the corresponding non-medicated formulations, the percentage of cell survival in the two studied human cell lines (HepG2 and MCF-7 Cells) was determined. Cells were treated with 3.125, 6.25, 12.5, 25, and 50 µg/mL doses for each formulation (n = 3). At higher drug doses of 25 and 50 µg/mL, the cell viability percentage for both cell models dramatically decreased to less than 10%. Accordingly, concentrations higher than 12.5 µg/mL were excluded as they produced almost complete cell death. Formulations F2, F3, F5, and F6 showed higher anticancer activities than their corresponding non-medicated formulations, as depicted in Table 2 and Figure 2. This finding demonstrates that SNEDDS has higher cellular permeability, which can be explained based on the rapid internalization of its component via fluid-phase pinocytosis, in contrast to 6-MP, which crosses cell membranes via passive diffusion. Furthermore, the higher initial release of 6-MP and curcumin in SNEDDS demonstrated that 6-MP and curcumin are in a dissolved form. The high solubility of these drugs in SNEDDS may reduce HepG2 and MCF-7 cell viability compared to free 6-MP [30].

It has been reported that curcumin has anticancer activity against many types of cancer cell lines, such as the breast cancer model MCF-7 cells [31], which have positive estrogen and progesterone receptors and are poorly aggressive and invasive, and the MDA-mb231 cells that lack both receptors [32]. Curcumin inhibits and suppresses a variety of cancer cells’ ability to proliferate by upregulating p53 and other cancer-suppressive genes while downregulating anti-apoptotic gene products [11].

At a concentration of 12.5 µg/mL, formulations F1 and F4 (contain the lowest concentration of turmeric oil) reduced the growth of MCF-7 cells by about 49 and 41%, respectively and the growth of HepG2 cells by 46 and 48%, respectively. Their corresponding non-medicated formulations inhibited HepG2 cells by about 32 and 29%, respectively, and MCF-7 cells by about 37 and 27%, respectively. Higher growth inhibition (71–76%) of the HepG2 cell model was achieved by the other 6-MP-loaded SNEDDS formulations at a concentration of 12.5 µg/mL, as well as of the MCF-7 cell model (63–76%) at the same dose range (Table 2).

*Curcuma longa* active oil ingredients have been shown to suppress the growth of breast (SKBR-3), pancreatic (PANC-1), and prostate (CP-3) cancer cell lines. When paclitaxel was combined with the curcumin active ingredients, an enhanced effect was obtained in terms of the growth inhibition of the studied cancer cells [33]. Curcumin encapsulated in protein nanoparticles showed a better anticancer effect against MCF-7 [34]. The downregulation of MMP-2 (matrix metalloproteinase) and overexpression of TIMP-1 (tissue inhibitor of metalloproteinase), which ate two frequent effector molecules associated with controlling tumor cell invasion, appear to be the mechanisms through which these anti-invasive effects are achieved [35,36].

Formulations F1 and F4 and their corresponding non-medicated formulations (F7 and F10), which contain the minimum amount of turmeric oil (15%), were selected for further study as they showed noticeable cell inhibition. And, they were characterized by their smaller particle size, which will enable their permeation through the biological membrane (Table 1).

**Figure 3:**
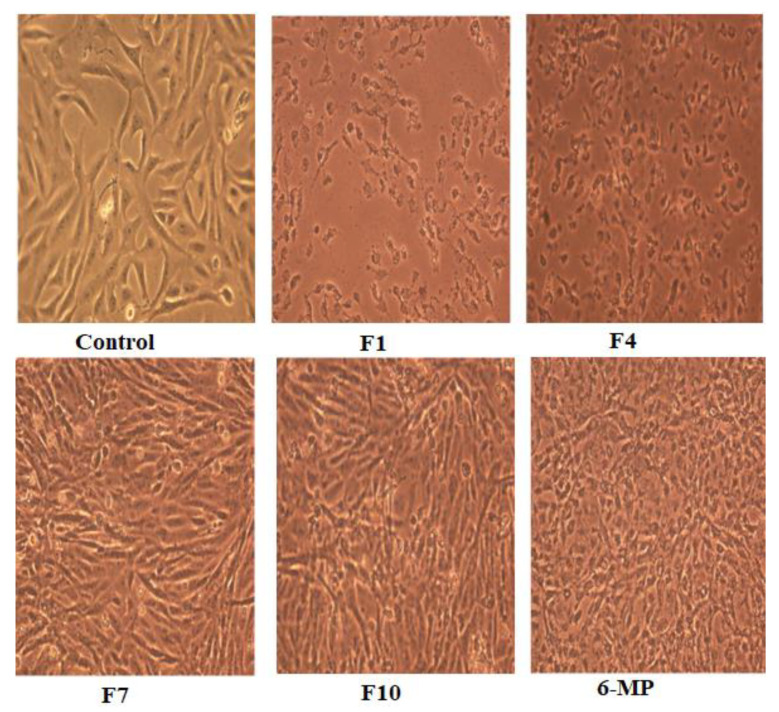
Morphology of HepG2 cells before (control) and after treatment with 12.5 μg/mL of the medicated SNEDDS (F1 and F4), the non-medicated equivalent formulation (F7 and F10), and 6-MP alone.

Figure 3 demonstrates the morphological changes in HepG2 cells. There was a significant change in the shape of the treated cells into spherical form after being treated with F1 and F4 loaded with 12.5 μg/mL. F1 treatment resulted in more condensed cells than F4, which were characterized by condensed cytoplasm and cellular shrinkage creating clusters. Additionally, HepG2 cells treated with non-medicated formula (F7 and F10) showed an irregular morphology. Although SNEDDS formulation was the most effective option for HepG2, 6-MP to HepG2 had more deformed cells and inconsistent shapes.

### 3.3. Apoptosis of HepG2 Treated with Pure 6-MP and SNEDDS Formulations

**Figure 4:**
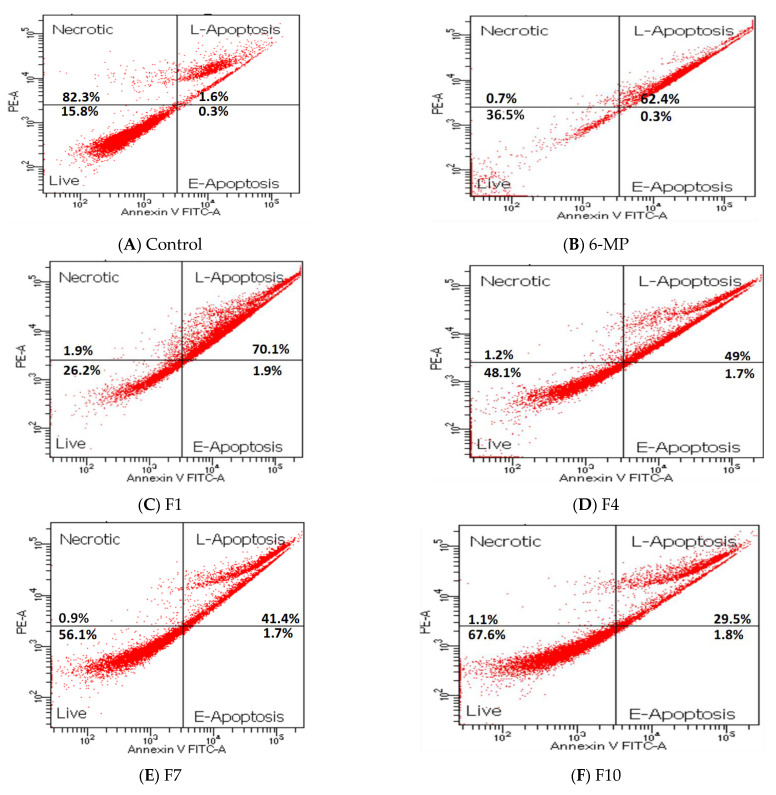
Necrosis percentage and early apoptosis of HepG2 cell line treated with pure 6-MP, drug-loaded SNEDDS (F1 and F4), and the corresponding non-medicated SNEDDS (F7 and F10) formulations. (**A**) Control; (**B**) 6-MP; (**C**) F1; (**D**) F4; (**E**) F7; (**F**) F10.

HepG2 cells were selected to study the apoptosis of the pure drug and the promising formulations. Cells treated with 25 µg/mL 6-MP and 12.5 µg/mL of F1, F4, F7, and F10 illustrated late apoptotic rates of 62.7, 72, 51, 43, and 31%, respectively. Among the studied SNEDDS formulations, F1 had the highest late apoptosis rate, while F10 showed the lowest rate. HepG2 cells treated with 6-MP, F1, F4, F7, and F10 showed almost no necrosis and early apoptosis (Table 3 and Figure 4). DNA condensation, fragmentation, and morphological alterations are the hallmarks of late apoptosis [37]. Pure 6-MP showed marked programmed cell death in a late state, and it was expected to inhibit cell growth, as previously reported [38]. Curcumin inhibited the phosphatidylinositol 3-kinase (PI3K)/protein kinase B (AKT) signaling pathway, which, in turn, prevented the proliferation of human non-small cell lung cancer cells (A549) and B-cell chronic lymphocytic leukemia (CLL-B) and induced death [39,40]. Conversion of 6-MP into turmeric oil-based SNEDDS formulation showed the minimum percentage cell viability and the highest percentage late apoptosis compared to the pure drug and the other SNEDDS formulations.

### 3.4. Cell Cycle Analysis of HepG2 Treated with Pure 6-MP and SNEDDS Formulations

**Figure 5:**
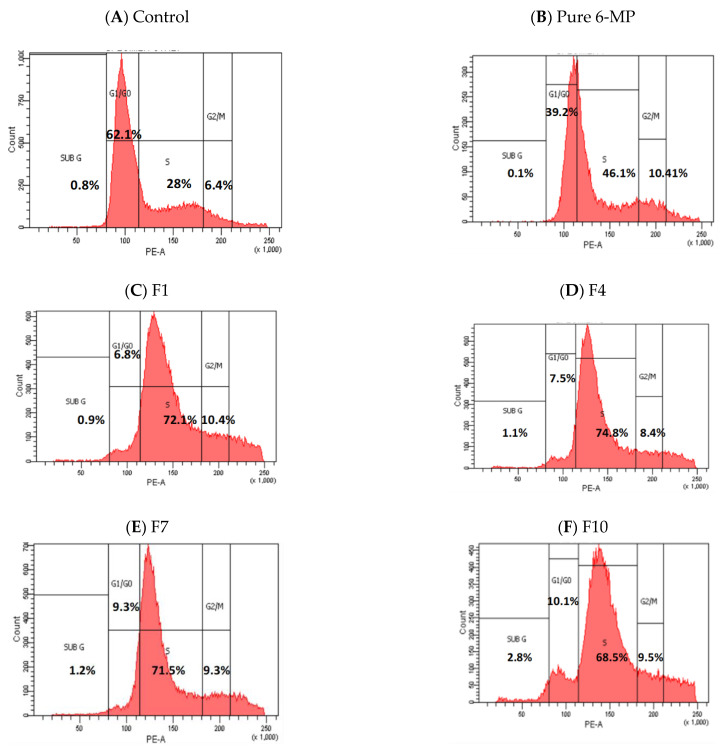
Pattern of the cell cycle for normal HepG2 cells (**A**) and cells treated with 6 MP (**B**), as well as the SNEDDS formulations F1 (**C**), F4 (**D**), F7 (**E**), and F10 (**F**).

**Figure 6:**
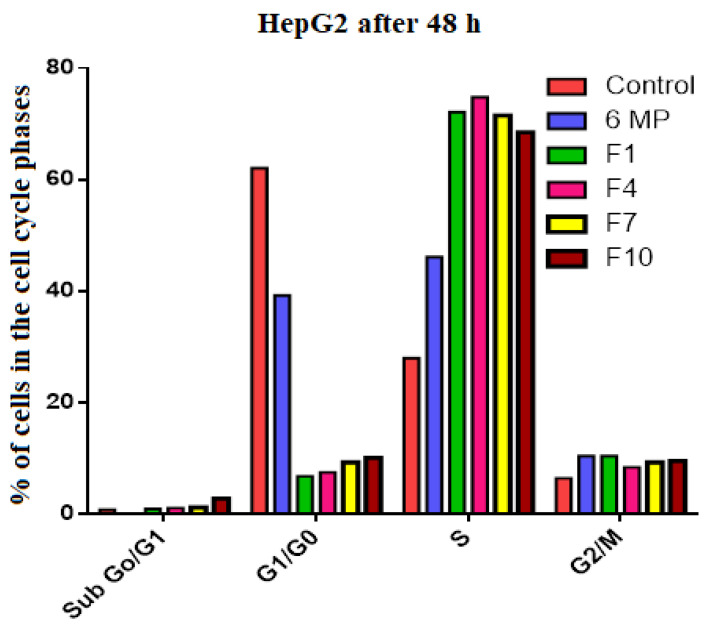
Percentage of HepG2 cells in the G0/G1, G1/Go, S, and G2/M phases for the control group and cells treated with Free 6 MP, F1, F4, F7, and F10.

HepG2 cells treated with 25 µg/mL of 6-MP and 12.5 µg/mL of F1, F4, F7, and F10 revealed that cells were halted in the S phases by 46, 72, 75, 72, and 69%, respectively, while the control “untreated” cell showed only 28% i.e., S-phase fold changes in cells treated with 6-MP, F1, F4, F7, and F10 relative to non-treated cells were 1.64, 2.58, 2.67, 2.55, and 2.44, respectively. When the HepG2 cells were treated with pure 6-MP and the studied SNEDDS formulations (F1, F4, F7, and F10), the proportion of cells in G1/G0 was considerably reduced, falling by 39, 6.8, 7.5, 9.3, and 10.1%, respectively, in comparison to untreated cells (62%). G1/G0-phase fold changes in cells treated with 6-MP, F1, F4, F7, and F10 were reduced by 1.58, 9.13, 8.33, 6.67, and 6.14, respectively, compared to non-treated cells (Table 4, Figure 5 and Figure 6). Cyclin A and Cyclin B were inhibited by curcumin. Cyclin A is a protein that triggers the S phase by interacting with cyclin-dependent kinase 6 (cdk6), which phosphorylates numerous DNA replication-related proteins. Cyclin B interacts with cdk1 to start mitosis in the M phase [41].

In human hepatoma G2 cells, curcumin caused DNA damage to both the mitochondrial and nuclear genomes. By boosting ROS production, lipid peroxidation, and DNA damage at high dosages, curcumin induced oxidative stress [42]. When curcumin was used to treat hepatoma cells, more ROS were produced, which impacted the enzyme histone acetyltransferase (HAT), which, in turn, regulates the level of histone acetylation. Curcumin specifically acted on ROS formation to generate a considerable reduction in histone acetylation in human hepatoma cells [43]. By reducing the expression of p21-Ras, p53, and NF-B, curcumin also demonstrated protection against diethyl nitrosamine (DENA)-induced hyperplasia and HCC in rodents [44].

In this work, we developed a turmeric-based SNEDDS formulation that can be used as a carrier for 6-MP to enhance the drug permeation via the nanocarrier system and boost the drug’s antiproliferative activities by incorporating turmeric oil. The formulations of the smallest particle size (F1 and F4) successfully achieved this goal.

## 4. Conclusions

Turmeric oil was extracted from the dried rhizomes of *Curcuma longa* and used in a concentration range of 15–35% to develop 6-MP-loaded turmeric oil-based self-nanoemulsifying drug delivery system (SNEDDS) formulations. The prepared formulations were measured in the nanosized range and showed good particle size distribution and high drug-loading efficiency. Low oil formulations with 15% turmeric oil loaded with 6-MP (F1 and F4) demonstrated smaller particle sizes and detectable antiproliferative efficacy against HepG2 and MCF-7 cells. Drug-loaded SNEDDS showed lower IC_50_ than the corresponding non-medicated SNEDDS and the pure 6-MP. HepG2 cells treated with the pure drug, the drug-loaded SNEDDS (F1, F4), and the corresponding non-medicated SNEDDS (F7 and F10) showed no necrosis or late apoptosis. The proportion of HepG2 cells in the G1/G0 phase was considerably reduced, being between 6.8 and 10.1% when treated with the SNEDDS formulations, compared to 62% for untreated cells. Accordingly, 6-MP loaded turmeric-based SNEDDS is a promising nanocarrier system that can enhance the drug’s antiproliferative activities.

## Figures and Tables

**Table 1:** Composition and characterization of the prepared 6-MP SNEDDS formulations.

Run	Composition			Characterization			
	TO	Tween 80	DMSO	Maximum Solubility	Particle Size	PDI	Zeta Potential
	(*w*/*v*)	(*w*/*v*)	(*w*/*v*)	(mg/mL)	(nm)		(mV)
F1	15	10	75	20	303.6 ± 19.3	0.431	−12.35 ± 0.12
F2	25	10	65	11.11	333.5 ± 9.7 *	0.429	−27.70 ± 0.46 *
F3	35	10	55	9.09	360.3 ± 6.2 *	0.692	−30.05 ± 0.07 *
F4	15	20	65	19.4	319.2 ± 39.3	0.662	−12.35 ± 0.15
F5	25	20	55	9.02	399.3 ± 4.0 *	0.461	−25.19 ± 0.16 *
F6	35	20	45	7.69	425.7 ± 7.4 *	0.401	−34.10 ± 0.06 *

Abbreviations: SNEDDS: self-nanoemulsifying drug delivery system, 6-MP: 6-mercaptopurine, TO: turmeric oil, DMSO: dimethyl sulfoxide, PDI: polydispersity index. * *p* < 0.001 versus F1.

**Table 2:** Percentage of HepG2 and MCF-7 cell viability after treatment with two different concentrations of free 6-MP: medicated and non-medicated SNEDDS formulations.

Code		HepG2 Cell Line				MCF-7 Cell Line		
	12.5 µg/mL	*p*-Value #	6.25 µg/mL	*p*-Value #	12.5 µg/mL	*p*-Value #	6.25 µg/mL	*p*-Value #
6-MP	55.70 ± 1.33		68.3 ± 4.09		62.53 ± 3.4		72.13 ± 1.2	
F1	53.68 ± 2.58	0.001715 *	76.32 ± 4.54	0.262352	51.24 ± 2.78	0.025611 *	71.53 ± 3.75	0.028023 *
F2	29.08 ± 1.44	0.023803 *	53.11 ± 2.64	0.000162 *	37.10 ± 4.33	0.274345	64.93 ± 6.34	0.050307
F3	27.03 ± 1.05	0.100611	42.8 ± 2.07	0.047353 *	23.36 ± 0.66	0.006331 *	56.11 ± 2.37	0.000836 *
F4	52.84 ± 3.46	0.006941 *	66.28 ± 4.64	0.010462 *	59.94 ± 1.67	0.003056 *	71.36 ± 4.8	0.080496
F5	24.50 ± 1.75	0.001281 *	39.2 ± 1.34	0.003569 *	26.78 ± 3.07	0.00497 *	35.13 ± 3.47	0.010065 *
F6	24.71 ± 0.17	0.001767 *	41.91 ± 3.17	0.006454 *	24.46 ± 2.36	0.095437 *	41.79 ± 2.29	0.286597
F7	68.32 ± 2.20		79.32 ± 1.63		63.42 ± 5.4		79.36 ± 1.45	
F8	38.20 ± 4.21		77.1 ± 1.45		42.43 ± 5.87		78.95 ± 6.06	
F9	30.17 ± 2.32		52.07 ± 5.26		30.31 ± 2.20		72.8 ± 2.16	
F10	71.30 ± 5.22		81.11 ± 3.23		73.45 ± 3.16		81.28 ± 5.59	
F11	39.75 ± 2.75		52.37 ± 3.45		46.84 ± 5.37		54.55 ± 6.45	
F12	37.89 ± 3.07		53.23 ± 2.00		30.144 ± 3.85		45.5 ± 4.67	

Abbreviations: HepG2: a human hepatoma cell line model, MCF-7: a breast cancer cell line model, 6-MP: 6-Mercaptopurine, F1–F6: the medicated self-nanoemulsifying drug delivery system formulations, F7–F12: the corresponding non-medicated self-nanoemulsifying drug delivery system formulations. # *p*-values formulation loaded 6-MP compared to non-medicated i.e., F1versus F7,F2 versus F8, F3 versus F9, F4 versus F10, F5 versus F11, F6 versus F12. * *p*-value < 0.05 is statistically significant.

**Table 3:** Percentage of necrosis and early apoptosis of HepG2 cell lines treated with pure 6-MP, drug-loaded SNEDDS (F1 and F4), and the corresponding non-medicated SNEDDS (F7 and F10) formulations.

Treatment	% Viable Cells	% Necrosis	% Early Apoptosis	% of Late Apoptosis	Fold of Changes in Late Apoptosis vs. Control	% Total Apoptosis
Control	82.3	1.6	0.3	15.8		16.1
6-MP	36.5	0.7	0.3	62.4	3.95	62.7
F1	26.2	1.9	1.9	70.1	4.43	72.0
F4	48.1	1.2	1.7	49.0	3.1	50.7
F7	56.1	0.9	1.7	41.4	2.6	43.1
F10	67.6	1.1	1.8	29.5	1.87	31.3

**Table 4:** The percentage of cells in the cell-cycle phase of HepG2 treated with Free 6 MP, F1, F4, F7, and F10.

	Control	6-MP	F1	F4	F7	F10
Sub G1	0.8	0.1	0.9	1.1	1.2	2.8
G1/G0	62.1	39.2	6.8	7.5	9.3	10.1
S	28.0	46.1	72.1	74.8	71.5	68.5
G2/M	6.4	10.4	10.4	8.4	9.3	9.5

## Data Availability

Not applicable.
